# Parafoveal X-masks interfere with foveal word recognition: evidence from fixation-related brain potentials

**DOI:** 10.3389/fnsys.2013.00033

**Published:** 2013-07-23

**Authors:** Florian Hutzler, Isabella Fuchs, Benjamin Gagl, Sarah Schuster, Fabio Richlan, Mario Braun, Stefan Hawelka

**Affiliations:** ^1^Department of Psychology, Centre for Neurocognitive Research, University of SalzburgSalzburg, Austria; ^2^Department of Basic Psychological Research and Research Methods, Faculty of Psychology, University of ViennaVienna, Austria

**Keywords:** visual word recognition, preview benefit, invisible boundary technique, parafoveal masks, eye movements, EEG

## Abstract

The boundary paradigm, in combination with parafoveal masks, is the main technique for studying parafoveal preprocessing during reading. The rationale is that the masks (e.g., strings of X's) prevent parafoveal preprocessing, but do not interfere with foveal processing. A recent study, however, raised doubts about the neutrality of parafoveal masks. In the present study, we explored this issue by means of fixation-related brain potentials (FRPs). Two FRP conditions presented rows of five words. The task of the participant was to judge whether the final word of a list was a “new” word, or whether it was a repeated (i.e., “old”) word. The critical manipulation was that the final word was X-masked during parafoveal preview in one condition, whereas another condition presented a valid preview of the word. In two additional event-related brain potential (ERP) conditions, the words were presented serially with no parafoveal preview available; in one of the conditions with a fixed timing, in the other word presentation was self-paced by the participants. Expectedly, the valid-preview FRP condition elicited the shortest processing times. Processing times did not differ between the two ERP conditions indicating that “cognitive readiness” during self-paced processing can be ruled out as an alternative explanation for differences in processing times between the ERP and the FRP conditions. The longest processing times were found in the X-mask FRP condition indicating that parafoveal X-masks interfere with foveal word recognition.

## Introduction

Most of what we know about parafoveal preprocessing is based on eye movement studies which administered the invisible boundary technique (Rayner, 1975). The boundary technique makes possible to experimentally manipulate the characteristics of the upcoming, parafoveal word. To illustrate, an invisible boundary is placed in a sentence before a target word. As long as the reader does not cross the boundary, the preview of the target words is experimentally manipulated (e.g., masked). When the reader's eyes cross the boundary, the preview is replaced by the target word.

Central to the present study is a variant of the boundary paradigm during which the parafoveal preview is masked. In this kind of experimentation, a parafoveal preview is presented which is either valid, that is, identical to the target word or partially valid (e.g., preview: *vievcn* or *viewXX*—target: *viewer*). The conditions with the valid and the partially valid previews are compared to a “baseline” condition in which the parafoveal preview of the target word is entirely masked. The masks, which are used most often, are either different letter masks (e.g., *nmovcn*—*viewer*) or of X-masks (e.g., *XXXXXX*—*viewer*; see Rayner, 2009). The rationale is that the mask prevents parafoveal preprocessing. The critical contrast is whether (and to what extent) participants are faster in the subsequent foveal recognition of the target word, when they are presented with (partially) valid previews compared to the baseline condition. If the processing times are shorter in the experimental condition than in the baseline-condition, then the standard interpretation is that useful information from the parafoveal preview was extracted during parafoveal preprocessing. This information may assist foveal processing of the target word. Put differently, the parafoveal preview facilitated foveal word recognition.

This interpretation, however, is crucially dependent on the “neutrality” of the mask, that is, the parafoveal mask itself must not induce uncalled-for effects during parafoveal preprocessing and must not affect (i.e., interfere with) foveal processing of the target word. If, to the contrary, the mask actually did affect parafoveal preprocessing and, in the most detrimental case, interfered with foveal processing as a consequence, then the interpretation sensu facilitation could be unwarranted. To illustrate, let us assume that a parafoveal mask (e.g., an X-mask) disrupts parafoveal processing and interferes with the subsequent foveal processing of the target word. As a consequence, the parafoveal preview of the mask may lead to a prolongation of foveal word recognition of, say, 30 ms. In such a case, an ostensible benefit of parafoveal preprocessing of, for example, a partially valid preview would be substantially overestimated, if it was derived from the contrast with the “baseline” condition.

Whether X-masks or different letter masks do indeed not elicit uncalled-for effects on foveal word recognition was seldom explicitly investigated. One exception is an early study by Rayner et al. (1978) whose finding led to a (short-lived) theoretical controversy about the suitability of various types of parafoveal masks (McClelland and O'Regan, 1981a,b; Rayner and Slowiaczek, 1981). To illustrate, Rayner and Slowiaczek (1981) reported that “*the direction of* […] *preview effects is crucially dependent on the choice of the baseline condition*” (p. 645). Thus, “*some kind of neutral preview must be found to assess costs and benefits of information extracted from parafoveal vision*” (McClelland and O'Regan, 1981b, p. 653). More recently, Jordan et al. (2003) pointedly stated that “[…] *in the absence of clear unequivocal evidence that a primary experimental manipulation does not produce secondary, unwanted influences, it is prudent for researchers to seek to minimize the potential for these experimental side effects*. […] *When the efficacy of a particular letter pair in word recognition is investigated, merely replacing all other letters in words with xs is unlikely to satisfy this principle of good scientific practice*” (p. 901).

These reservations about the application of parafoveal masks, however, had virtually no impact in the research field. The X-mask and the different letters mask are still the most common choice in eye movement studies on reading which use the boundary paradigm. Only recently, the issue of potential uncalled-for side effects of parafoveal masks was seized again. Kliegl et al. (2013) re-analyzed the data from a published eye movement study (McDonald, 2006) which used the boundary technique and different letter masks. A critical finding was that the masks elicited inflated gaze durations on the target word when the preceding fixation was in close proximity to the target word compared to instances where the preceding fixation was remote from the target word. The rationale of this comparison is that in case of near fixations the masks are perceived with high visual acuity (i.e., are more salient) compared to remote fixations. The inflated gaze duration for near fixations thus indicates that the masks interfered with foveal word recognition. Kliegl et al. concluded that preview effects, which were up to now subsumed under the umbrella term “preview benefit,” could actually be a complex mixture of benefits and costs.

The objective of the present study was to assess the effect of the parafoveal preview of X-masks on the subsequent foveal processing of words. In particular, we were interested in the time course of the effect of the parafoveal mask. To this end, we combined eye movement recording and EEG—two methods which both provide high temporal resolution. By combining these methods one can obtain fixation-related brain potentials (FRPs; Baccino and Manunta, 2005; Hutzler et al., 2007; Dimigen et al., 2011). The technique makes possible to assess cognitive processes in an experimental setting which permits parafoveal preprocessing (of experimentally manipulated previews). Thus, the technique provides the possibility to measure the temporal dynamics of visual word recognition in a relatively natural (and hence ecologically valid) setting. Monitoring the eye movements granted the participants to read (more specifically, to parafoveally preview and foveally process) the words at their individual reading speed. The concurrently recorded EEG allowed the assessment of the temporal dynamics of visual word recognition after previewing an X-mask compared to preprocessing a valid preview.

To assess the effect of previewing an X-mask on the subsequent foveal word recognition we relied on an established effect from the event-related brain potential (ERP) literature, that is, the old/new effect in a continuous recognition task (Friedman, 1990) which was successfully used in a previous FRP study (Hutzler et al., 2007). The participants were presented with a list of 5 words and they had to judge whether the 5th word was the same as one of the previous 4 words, or whether it was a new word. In standard ERP conditions, in which the words are serially presented one-by-one, the task reliably elicited more positive waveforms for “old” words than for “new” words about 250 ms after stimulus onset particularly for electrodes at central/parietal sites of the scalp (Friedman, 1990) and it was shown that the effect is more pronounced at electrodes over the right than over the left hemisphere (Hutzler et al., 2007).

**Figure 1:**
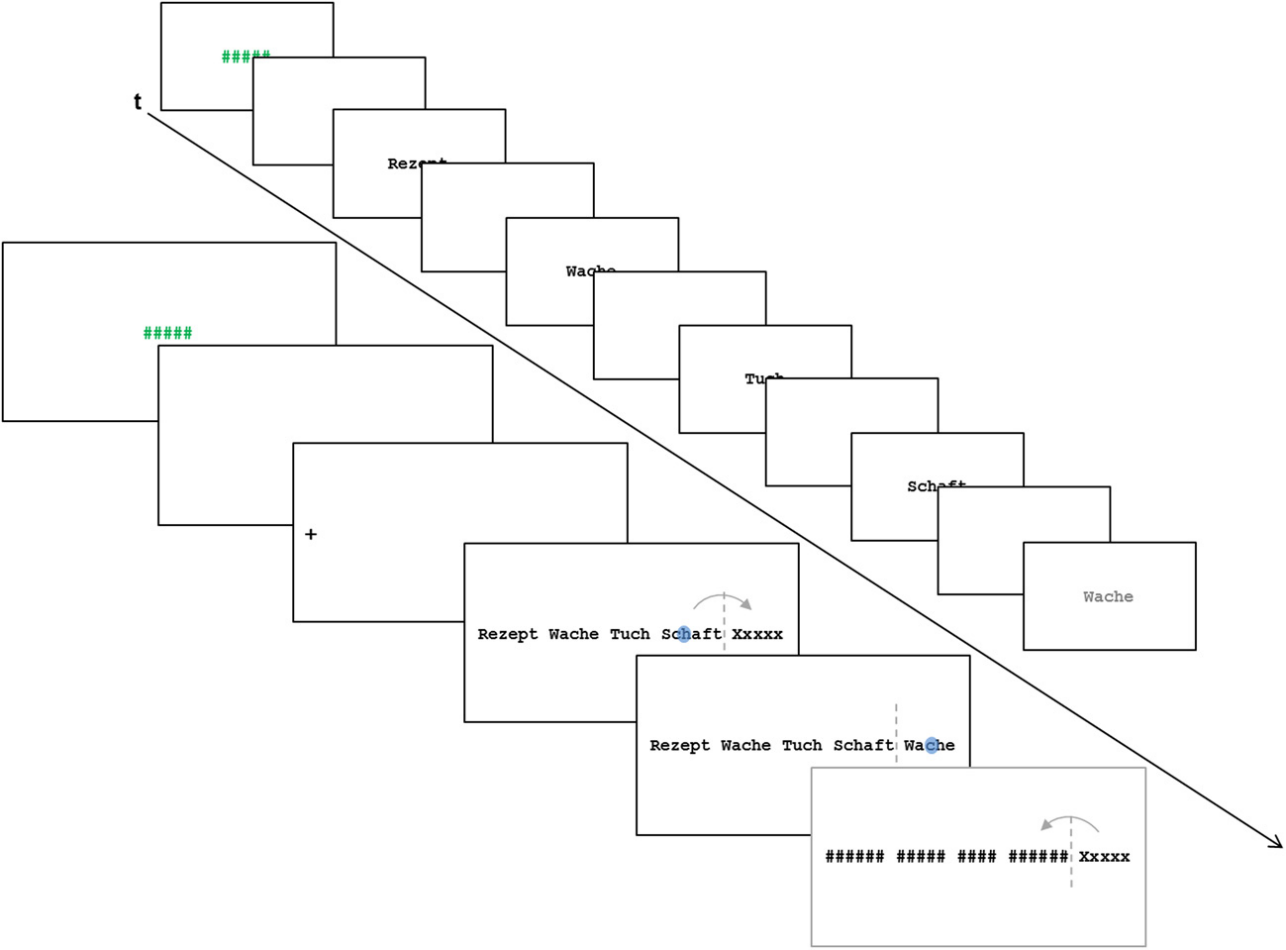
**Schematic illustration of the events in a trial of the X-mask FRP condition and a trial of the ERP conditions.** The blue dot in the illustration of the FRP condition represents a fixation; the arrow represents a saccade which crosses the invisible boundary (dashed line) before the target word. In the FRP conditions, the time-points for averaging the FRPs were the start of the first fixation on the target words. In the ERP conditions, the appearance of the target words was the critical event for averaging the ERPs. The final screen in the FRP condition was only presented in case of a regression from the target word toward the preceding words and served to dissuade the participants from regressions.

In the present study, we administered two FRP conditions. Both conditions presented rows of unrelated words. One condition permitted parafoveal preprocessing by presenting valid previews of the target words. In the other condition, the target words were X-masked until fixation (to be precise, until crossing the invisible boundary before the target word). In addition to the two FRP conditions, we administered two ERP conditions in which the words were presented serially (i.e., in isolation one-by-one). In one of these conditions (i.e., the fixed-pace condition) the words were presented with a fixed, unvarying timing. In the other (the self-paced) condition, the presentation of the words were manually triggered by the participants. Figure 1 depicts the events of a trial of the X-mask FRP condition and of a trial of the ERP conditions.

The start of a significant divergence of the FRP and ERP curves in response to the experimental conditions, that is, the onset of the old/new effect, is considered as the earliest point in time of differences in processing the target words (henceforth processing time). We expect that the valid-preview FRP condition will elicit the shortest processing times of the target words due to parafoveal preprocessing (i.e., a preview benefit). Theoretically relevant is the comparison of the processing times in the X-mask FRP condition with the processing times in the ERP conditions. If processing times are prolonged in the X-mask FRP condition compared to the ERP conditions, then this would indicate interference of the X-masks with foveal word recognition. Comparing the fixed-pace ERP condition with the self-paced ERP condition serves to assess whether “cognitive readiness” during self-paced processing account for differences in processing times between the fixed-pace ERP condition and the two (inherently self-paced) FRP conditions.

## Methods

### Participants

Fifteen native German-speaking right-handed students (11 females) of the University of Salzburg (mean age 24 years) with normal or corrected-to-normal vision participated in the study.

### Procedure

To estimate the time-course of visual word recognition, we used the same marker-effect as in Hutzler et al. (2007), that is, the old/new effect in a continuous recognition task (Friedman, 1990). Figure 1 schematically depicts the events of a trial from an FRP and a trial from the ERP conditions. In all four settings (fixed-pace ERP, self-paced ERP, valid-preview FRP, and X-masked FRP), five unrelated words were presented and participants had to indicate via button press whether the 5th word (henceforth: target word) was the same as any of the four previously encountered words (“old” trial) or not (“new” trial). Trials in the old-condition consisted of three filler words, one word which was the same as the target word and the target word. The word which was the same as the target word was at the 1st, the 2nd, or the 3rd position of the word list (counterbalanced across trials), but was never at the 4th position (i.e., it never was the pre-target word). The trials in the new-condition consisted of four filler words and a not previously presented word in the 5th position. Each of the four experimental setups presented 100 trials (50 “old” and 50 “new” trials) resulting in a total of 400 trials.

All words were nouns ranging in word length from 3 to 8 letters. As evident from Figure 1, the words all had a capitalized first letter which is the correct form for German nouns. Words were presented in Courier New on a white background. The target words of the four experimental setups and the two conditions (old vs. new) were selected in such a way that they were closely matched on 8 word characteristics across setups and conditions (see Table A1). Furthermore, the pre-target words were matched on six characteristics (Table A2) in order to hold constant the processing difficulties imposed by these words (i.e., the foveal load; Henderson and Ferreira, 1990). The rigorous matching precludes that differences in findings across experimental setups and conditions are due to differences in the characteristics of the target words or due to spillover effects from the pretarget words.

In all settings, each trial started with the presentation of a string of five hashes (#; varying between 1500 and 3000 ms to prevent phase locking on trial timing). The hash string signaled the participants that they were allowed to blink. Thereafter, a blank screen was presented for 2000 ms. The target words (i.e., the 5th word) remained on the screen until the participants indicated with a button press (with their index fingers on a gamepad) whether it was an “old” or a “new” word. The mode of response (*old word—left button*; *new word—right button*) was reversed after the presentation of half of the trials in each condition (to: *old word—right button*; *new word—left button*). The participants were required to respond as accurately as possible, but speed was not emphasized. The sequence of the experimental setups was one of the FRP conditions followed by one of the ERP conditions, followed by the other FRP and then the other ERP condition or vice versa.

#### ERP settings

The five words of a trial were presented singly and serially (i.e., word by word) at the center of the screen. The first four words of a trial were presented in black color. The target word, in contrast, was dark gray, allowing the participants to identify the 5th word as the target. In the fixed-pace ERP setting, the words were presented for 800 ms, one after another with a 500 ms blank screen in-between. In the self-paced ERP setting, the words remained on the screen until the participants pressed a button (with their thumb) and was then followed by a blank screen (200 ms). The intertrial-interval (blank screen) was 2000 ms. Three practice trials preceded the experimental conditions.

#### FRP settings

At the beginning of a trial, a fixation cross was presented left of the screen center. Participants were required to fixate the cross and after the eye tracker registered the fixation, a blank screen was presented for 200 ms. (If the eye tracking system did not detect a fixation on the fixation cross within 5 s, the eye tracker was re-calibrated, see below). After the fixation-check, the five words of a trial were presented simultaneously in a row in such a way that the participants now fixated the first letter of the first word of the list. The series of words remained on the screen until response. In the X-mask preview condition, the target word was X-masked until the participants crossed the invisible boundary between the target word and the preceding word. The other condition presented a valid preview of the target word. To dissuade the participants from regressing back from the target word to the preceding words, we again applied the boundary technique. The first fixation on the target word reactivated the boundary between the target word and the 4th word. If the participant made a regression toward the preceding words (and in so doing crossed the boundary) all preceding words were replaced with hash-mark strings. Such trials were omitted from analyses. Ten practice trials preceded the experimental conditions.

### Apparatus

Multichannel EEG was recorded from 32 Ag/AgCl electrodes mounted with a modular elastic cap (Easy Cap, Falk-Minow Systems, Germany) on standard positions according to the 10–20 system. Scalp electrodes were recorded referentially against linked earlobes (as common reference) with a sampling rate of 1000 Hz. To monitor horizontal and vertical eye movements, EOG was recorded bipolar from the outer canthus of each eye as well as from below and above the right eye (recorded bipolarly against FC1). Signals were amplified using a 32 channel Brainamp (BrainProducts, Germany) amplifier with a 0.1–1000 Hz band pass and a 50 Hz notch filter. Impedances for scalp electrodes were kept below 5 k. Eye movements were recorded (monocular for the right eye) with an EyeLink CL tower mount eye tracker (SR Research, Ontario, Canada) with a sampling rate of 1000 Hz. Before each of the two FRP conditions the eye tracker was calibrated with a horizontal 3-point calibration routine. The criterium for a successful calibration was an average tracking error of less than 0.5° of visual angle (max = 0.36° and 0.44° for the valid preview and the X-mask preview FRP conditions, respectively; *M* = 0.19 for both conditions). The calibration of the eye tracker was repeated, when the fixation control at the beginning of a trial failed (see above).

Participants sat at a viewing distance of 52 cm (held constant by a forehead and a chin rest) from a 21″CRT monitor. From the distance, a single letter of the words had a width corresponding to approx. 0.4° of visual angle. The monitor had a resolution of 1024 × 768 pixels and refreshed with 120 Hz. Stimulus presentation was controlled by the Experiment Builder software (SR Research Ltd., Canada). In the ERP settings, the point-in-time of the stimulus presentation was registered by the EEG recording equipment via standard communication (i.e., via the parallel ports of the Display PC and the EEG recorder). In the FRP settings, the point-in-time of the start of the first fixation on the target word was registered by the eye tracking system and sent to the EEG recorder. This point-in-time was corrected offline for the latency of the fixation detection algorithm of the eye tracking system. The default latency of the fixation detection algorithm is 36 ms and this value was fairly constant (in 97% of the instances it was either 36 or 37 ms). The value was never greater than 40 ms.

### Analysis

EEG data was analyzed using the EEG-Lab v6.01b toolbox (Delorme and Makeig, 2004) toolbox for MATLAB v7.0 (Mathworks, Natick, MA). For the analysis of ERPs and FRPs, the continuous EEG data was segmented upon the point-in-time at which the theoretically relevant target word (i.e., the 5th word of a trial) appeared (in the ERP settings) or when it was first-time fixated (in the FRP settings). EEG data was segmented from 100 ms before to 600 ms after these time points. Trials corrupted by eye blinks or EEG-artifacts (determined by visual inspection) were excluded from further analysis. The 100 ms interval prior to the appearance of the target words (ERP) or the first fixation on the target words (FRP) was used for baseline correction.

#### Artifact correction

EEG-artifacts due to horizontal eye movements were corrected by means of independent component analysis (ICA; Vigário, 1997). The ICA separates waveforms into components that are maximally independent from each other. The ICA component resembling the typical activity pattern and component map of horizontal eye movements is then removed prior to back-projection (see also Delorme et al., 2007). ICA proved to be useful for the identification and elimination of the horizontal eye movement artifacts typical for reading in previous studies (e.g., Hutzler et al., 2007, 2008; Plöchl et al., 2012). Subsequently, epochs were filtered with a 30-Hz low-pass filter.

## Results

Trials with incorrect responses were excluded from analysis (6 and 9% for ERPs and FRPs, respectively). The group means of the median response times of the participants were 887 and 962 ms in the valid and the X-masked FRP condition, and 955 and 1016 ms in the fixed-pace and the self-paced ERP condition, respectively. The analysis of response times by means of a 2 × 4 repeated-measures ANOVA with old vs. new words and condition (valid preview and X-mask FRPs, fixed-pace and self-paced ERPs) as within-subject factor revealed a significant main effect of condition; *F*_(3, 42)_ = 3.78, *p* < 0.05, a significant interaction between condition and old vs. new words; *F*_(3, 42)_ = 4.91, *p* < 0.01; but no main effect of old vs. new words, *F* < 1. *Post-hoc* comparisons, however, failed to reveal reliable differences between the four conditions. Concerning the old/new effect, response times were around 73 ms slower for old compared to new words in the X-masked FRP condition (*M* = 1002 and 929 ms, respectively; *p* < 0.05), but around 57 ms faster for old compared to new words in the self-paced ERP condition (*M* = 990 and 1047 ms; *p* < 0.01). No reliable differences between RTs in response to old vs. new words were found in the valid preview FRP and fixed pace ERP conditions.

**Figure 2:**
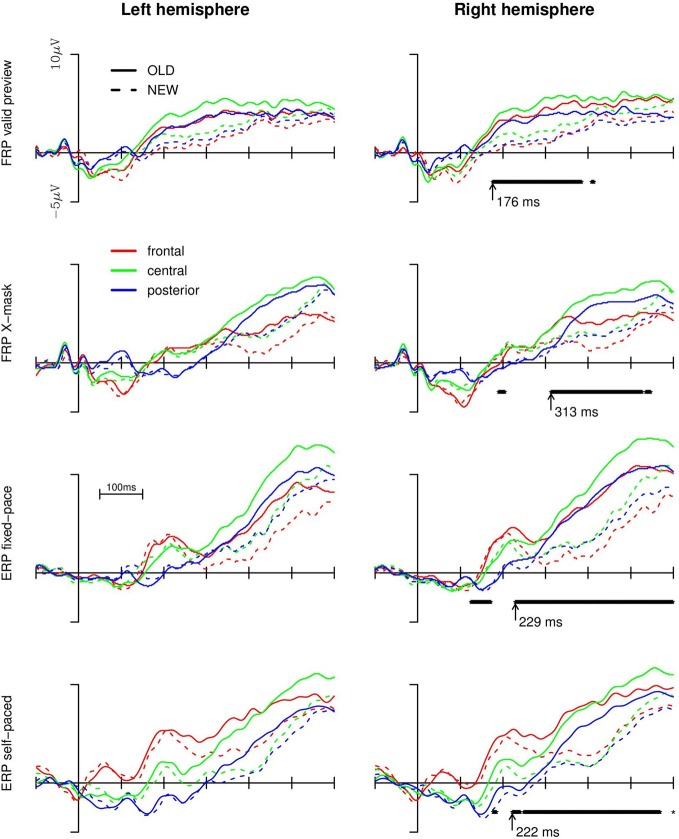
**Brain potentials (upper two rows: fixation-related; lower two rows: event-related) in the four experimental conditions for the left and right hemisphere and the old vs. new words.** A continuously reliable main effect of old vs. new words or the interactions of the effect with region or hemisphere (as revealed by point-by-point ANOVAs) are depicted below the FRP/ERP curves of the **right panel**. The arrows denote the earliest time point of the onset of the old/new effect (i.e., main effect or interaction).

For the statistical analysis of the old/new effect on the brain potentials we collapsed the data from electrode clusters in the left anterior (F3, FC1, FC5), central (C3, CP1, CP5), and posterior (P3, P7, T7) regions and for the corresponding electrodes of the right hemisphere (i.e., F4, FC2, FC6 and C4, CP2, CP6 and P4, P8, T8, respectively). For a first exploratory analyses, we submitted the averaged data from the regions to point-by-point repeated measures ANOVAs (i.e., for every 1 ms of the data stream) with old vs. new words, region (frontal, central, posterior), and hemisphere (left vs. right) as within-subject factors separately for each experimental condition. The ERPs and FRPs are depicted in Figure 2. In the right panel of the Figure we additionally depicted the time-points for which the ANOVA revealed continuously significant effects (*p* < 0.05) of old vs. new words, that is, either a main effect of old vs. new words or interactions of hemisphere and/or region with the factor old vs. new word (short-lived effects prior to 100 ms are not shown). The values beside the arrows indicate the onsets of such a continuously significant effect.

As evident from Figure 2, both FRPs and ERPs were more positive-going in response to old words compared to new words (from, dependent on the experimental setup, about 180 to 280 ms onwards). The effect was most pronounced for the central and anterior regions and more so in the right than in the left hemisphere (replicating previous findings with ERPs; Friedman, 1990; and ERPs and FRPs; Hutzler et al., 2007). For the valid preview FRP condition, the point-by-point ANOVAs revealed that the effect of old vs. new words began to be continuously significant from 176 ms after the start of the first fixation on the target word onward (until approx. 390 ms after the start of the first fixation). In the X-mask preview condition, no long-lasting, continuously reliable difference between old and new words (neither a main effect nor an interaction with region or hemisphere) emerged until 313 ms. Thus, the old/new effect emerged about 130 ms later in case of an X-mask preview compared to the valid preview of the target word. In the ERP conditions, the divergence points of the curves were intermediate and there was virtually no difference in the time points of the emergence of the old/new effect between the fixed-pace and the self-paced condition (i.e., at 229 and 222 ms, respectively). These time-points are about 50 ms later than the divergence point of the old/new effect in the valid preview condition, but they are considerably earlier than in the X-mask FRP condition.

Determining the temporal onset of the old/new effect individually for each participant would have been the prerequisite for a classical, inferential analysis of the differences in the onset of the old/new effect for the four conditions. The low signal-to-noise ratio of the EEG, however, did not allow such single-subject analyses. However, to assess the significance of the differences in the time courses of the old/new effect we administered a statistical analyses based on the *jackknife* procedure introduced by Ulrich and Miller (2001). This procedure makes possible to assess differences in the time-points of the emergence of an effect by a bootstrap procedure. In essence, the emergence of an effect is repeatedly assessed in subsamples of the original sample by consecutively leaving one subject out of the analyses (resulting, for the present analyses, in 15 subsamples with *n* = 14 for each subsample). The time-points of the subsamples are then submitted to a standard ANOVA. This procedure (to be specific, the use of subsample scores) leads to an underestimation of the error term of the ANOVA and hence the *F*-values associated with an effect (in the present case, the old/new main effect) must be corrected. The correction is administered by dividing the *F*-value(s) from the ANOVA by the squared number of subsamples minus 1 [i.e., *F*_*C*_ = *F*/(*n* − 1)^2^]. *Post-hoc* comparisons (with the Scheffé test in the present analysis) can also be carried out after correcting for the deflated error term. For details and the proof of the applicability of the procedure see Ulrich and Miller (2001; also Miller et al., 1998).

**Figure 3:**
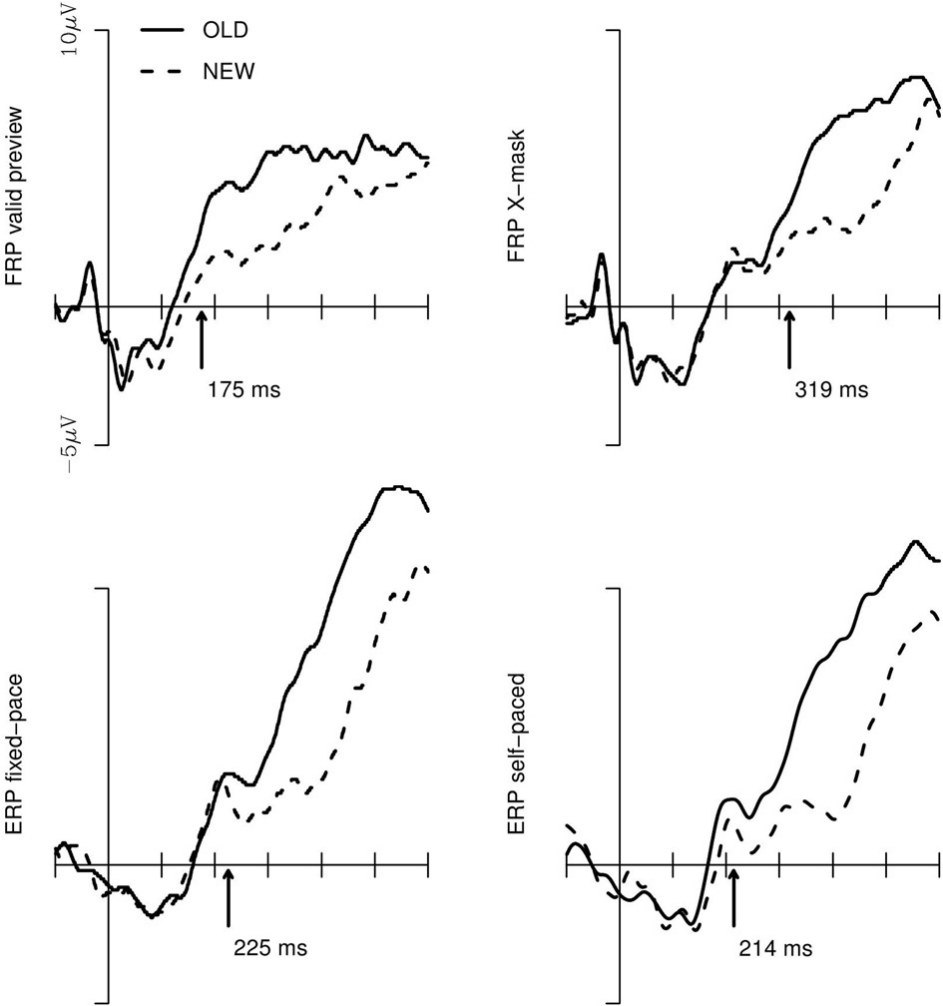
**Brain potentials in response to “old” vs. “new” words for the electrodes of the central cluster of the right hemisphere for the valid preview and the X-mask preview FRP conditions (upper row) and the fixed-pace and self-paced ERP conditions.** The arrows indicate the point-in-time of the emergence of the old/new main effect which was assessed with the jackknife procedure (Ulrich and Miller, 2001; see text).

The old/new effect is, as aforementioned, most pronounced at central electrodes in the right hemisphere (e.g., Friedman, 1990; Hutzler et al., 2007). Thus, we analysed the significance of the old/new effect for the respective cluster. Figure 3 depicts the FRPs and ERPs of this cluster. Furthermore, the Figure shows the onset of the old/new main effect provided by the *jackknife* procedure. The onsets represent, for each of the 4 experimental conditions, the mean of the first sample points of a sequence of a minimum of 30 sample points for which a one-sided, paired-sample *t*-test revealed a continuously and significantly higher amplitude of the FRP/ERP-curves in response to old vs. new words in the 15 jackknife-subsamples. The ANOVA with these subsample scores as the dependent measure and the 4 experimental setups as within-subject factor revealed that the old/new main effect differed significantly between the conditions; *F*_*C*(3, 42)_ = 11.4; *p* < 0.001. For the *post-hoc* Scheffè tests, the critical difference for the onsets of the old/new effect is 76 ms (corrected for the subsample-based error term and for a significance level of *p* < 0.05). Thus, the difference in the time points of the old/new effect was not reliable for the valid preview FRP condition compared to the two ERP conditions. Critically, the emergence of the old/new effect in the X-masked FRP condition was significantly delayed compared to all other conditions.

## Discussion

The aim of the present study was to explore, whether an X-mask, which is commonly used to mask a target word in the invisible boundary paradigm, interferes with the foveal processing of a target word. We administered, in the standard setting of the invisible boundary paradigm, FRPs for determining the relative time-course of word recognition processes at the highest possible temporal resolution. Additional event-related (ERP) setups assessed the time-course of word recognition without parafoveal preprocessing. The shortest processing times (around 180 ms) of the target words were observed, when a valid parafoveal preview was available in the FRP setting. As expected, when parafoveal preprocessing was prevented by an X-mask in the FRP setting, processing times were substantially prolonged. In the ERP conditions, the point-in-time of the old/new effect occurred substantially earlier than in the X-mask FRP condition.

The standard (i.e., fixed-pace) ERP condition and the FRP conditions did not only differ in the provision of parafoveal information. Another critical difference of the FRP conditions was that the acquisition of information was controlled by the participants themselves (i.e., internally; they moved their eyes when they were “ready” for processing the next word), whereas in the standard ERP setting the participants had no control of the acquisition of information. Thus, we reasoned that different findings of the ERP condition and the FRP conditions could (partially) reflect this difference, rather than (solely) the difference in the availability of parafoveal information. The self-paced ERP condition was administered to control for this possibility. However, the processing times were similar for the fixed-pace and the self-paced ERP conditions (around 220 ms) and hence we can discount the possibility that self-paced processing accounts for differences between the experimental setups.

The faster processing in case of valid previews compared to invalid previews clearly demonstrates that the marker effect of the present study (i.e., the old/new effect) was a suitable choice for assessing parafoveal preprocessing, in general, and the benefits and costs of the parafoveal previews, in particular. Parafoveal preprocessing is one of the key mechanism which enables fluent reading. It is, however, not the only mechanism. In case of natural texts, sentence level processing, such as inferring upcoming words from the preceding sentence context (i.e., the effect of word predictability) is also conducive for fluent reading. The objective of the present study, however, was to assess the “pure” (word-level) effect of previewing an X-mask vs. a valid preview. We reasoned that the presentation of lists of unrelated words revealed the pristine effect of the parafoveal X-masks, uninfluenced by effects of word predictability or contextual constraints.

The observed magnitude of the preview benefit in the valid preview FRP condition compared to the X-masked FRP condition of about 130 ms is surprisingly large in absolute terms. This prompts the question, whether this difference solely reflects preview benefits (to be attributed to the valid preview condition), or whether this difference might additionally reflect processing costs (due to interference in the X-mask condition). Comparing the X-mask FRP condition to the ERP conditions (which provided no parafoveal information) reveals that the latter interpretation is warranted. In the X-mask FRP condition, processing is substantially delayed (approx. 60 ms) compared to the ERP conditions.

The present finding suggests that an X-mask is not neutral, but interferes with the processing of the target word. The existence of a parafoveal preview benefit during reading is undisputed. Another question is, however, which particular processes are induced by the application of parafoveal masks. The present findings corroborates Kliegl et al.'s (2013) notion that by the application of parafoveal masks we probably assess a complex mixture of benefits and costs. Kliegl et al.'s study concerned different-letter masks, whereas the present study presented X-masks. The outcome, however, concur. Both studies indicate that parafoveal masks inflict processing costs on the subsequent recognition of the target word. The requirement for a baseline condition, that is, neutrality with regard to the theoretically relevant effect, is thus not fulfilled.

The implication of the findings is that processing benefits of a (partially) valid preview, are overestimated, when the estimate is derived from a baseline condition which presented parafoveal masks. Furthermore, it could be that ostensible preview benefits (of small magnitude) may do not reflect facilitation at all. To illustrate, Inhoff (1989) investigated, whether the final letters of a parafoveal word facilitate its subsequent recognition. The study revealed that preview “benefits” depended on the type of the baseline condition. The application of X-masks indicated a processing benefit for previewing the final letters of an upcoming word. Another condition used different letter masks and did not reveal such a benefit. In the light of the novel findings, which suggest that parafoveal masks interfere with foveal processing, it could be that Inhoff observed processing costs in the X-mask “baseline” condition, and not processing benefits in the valid preview condition. This is an issue which deserves further investigations with a proper baseline condition.

Interpreting the present findings as reflecting processing costs inflicted by a suboptimal baseline condition (i.e., a parafoveal mask) could be countered by the following argument: Processing costs due to invalid information necessarily imply processing benefits due to valid information. Put differently, if masking of, for example, the final letters of an upcoming word (e.g., *viewXX*) is thought to interfere with the subsequent recognition of the word, should not, in turn, the valid preview of the final letters (i.e., *viewer*) facilitate the recognition of the target word? This is not necessarily the case, if the abstraction level of the information which is extracted from the parafovea is taken into account: The interference of parafoveal masks can, in principle, occur at various different levels, from low-level visual information up to orthographic information. To illustrate, a parafoveal mask could interfere with the establishment of the correct visual representation of the target word (once fixated), because some low-level visual representation has to be amended or overwritten. The existence of such a low-level visual representation, however, is not necessarily on an orthographic level. It can be that some low-level visual representation of a parafoveal preview is established, but orthographic (or phonological, morphological, etc.) representations are not yet activated. In the foregoing example of the parafoveal preview *viewXX*, the *XX* might deter establishing a valid visual representation of the correct word, when it is fixated. Such a low-level visual interference, however, does not imply that the orthographic information of these final letters (e.g., abstract letter codes) is processed. In this case, a valid preview of the final letters does not necessarily provide discernible processing benefits, although a parafoveal mask at the same location may result in processing costs.

In the light of the present findings [and those by Kliegl et al. (2013)] it will be a crucial issue for future research to discover a neutral baseline-condition. Note that the problem is not limited to the issue of parafoveal processing (during reading), but is a general problem in all domains which conduct baseline-conditions in order to estimate the size (or the direction) of a theoretically relevant effect (e.g., in priming studies; Jonides and Mack, 1984). Thus, a solution for the problem developed in other domains could also be suitable to address the baseline-problem in studies on parafoveal preprocessing (and the boundary paradigm). Jacobs et al. (1995) presented an ingenious solution for priming studies (on visual word recognition), that is, the *incremental priming technique*. The technique provides a within-condition baseline which makes possible to use an experimental condition as a baseline with respect to itself. The logic is as follows: The informational value, that is, the salience of the primes is gradually increased (in steps from low salience toward full salience). In Jacobs et al. (1995) the salience was manipulated by varying the brightness of the primes. The critical aspect then is, how the processing times of the targets change in response to the increasing salience of the prime. If increasing salience speeds up target processing, then the prime is facilitatory. If, to the contrary, increasing salience prolongs target processing, then the prime interferes with processing. Thus, the critical advantage of the incremental priming technique is that an experimental condition is sufficient in itself for the examination, whether a specific type of information facilitates or interferes with processing. It is conceivable that the same logic can be applied for manipulating parafoveal previews (in combination with the boundary paradigm). The salience of a parafoveal preview could be varied in several ways such as varying the brightness/contrast of the parafoveal preview or visually degrading the preview by blurring or replacing pixels in the bitmap of the preview. We are currently testing several of these alternatives and the preliminary findings are promising.

To conclude, recent evidence justifies skepticism concerning the adequacy (more specifically, the neutrality) of those parafoveal masks which have been used most frequently in combination with the boundary paradigm. Different letter masks (Kliegl et al., 2013) as well as X-masks (the present study) inflict processing costs, that is, they interfere with foveal processing of the target words. These findings demonstrate that Rayner and McClelland debated in the early 1980's with good case over the question about a proper baseline. This debate, however, was a (mostly) theoretical discourse. Rayner and colleagues (back in 1981) already stated that this issue must be resolved empirically. However, the issue was not approached empirically for now more than 30 years. Thus, the jury is still out: On the one hand, on the issue of an adequate baseline condition for investigating preview benefits; on the other hand, on the validity of ostensible preview benefits which were inferred from contrasting valid previews with the previews of masks.

### Conflict of interest statement

The authors declare that the research was conducted in the absence of any commercial or financial relationships that could be construed as a potential conflict of interest.

## Appendix

**Table A1:** **Means (standard deviations) of the characteristics of the “old” vs. “new” target words separately for each of the four experimental setups**.

	**Fixed-paced ERPs**		**Self-paced ERPs**		**Valid-preview FRPs**		**Masked FRPs**	
	**Old**	**New**	**Old**	**New**	**Old**	**New**	**Old**	**New**
	***M* (*SD*)**	***M* (*SD*)**	***M* (*SD*)**	***M* (*SD*)**	***M* (*SD*)**	***M* (*SD*)**	***M* (*SD*)**	***M* (*SD*)**
Frequency^a^	31 (32)	30 (35)	30 (31)	31 (33)	31 (33)	31 (36)	30 (38)	31 (66)
Bigram count [N]^b^	580 (493)	577 (557)	582 (469)	577 (581)	577 (447)	579 (570)	579 (593)	580 (456)
Bigram freq.^c^	7576 (7293)	7492 (7868)	7541 (7004)	7559 (9086)	7529 (7807)	7556 (9060)	7488 (16668)	7525 (8018)
Syllables [N]	1.8 (0.4)	1.8 (0.4)	1.8 (0.4)	1.8 (0.4)	1.8 (0.4)	1.8 (0.4)	1.8 (0.4)	1.8 (0.4)
Letters [N]	5.68 (1.38)	5.68 (1.1)	5.68 (1.1)	5.68 (0.94)	5.68 (1.35)	5.68 (1.11)	5.68 (1.24)	5.68 (1.04)
Neighbors [N]^d^	1.98 (2.38)	1.98 (3.02)	1.98 (2.06)	1.98 (2.23)	1.98 (2.31)	1.98 (2.08)	1.98 (2.25)	1.98 (2.35)
Emotional valence^e^	0.2 (1.33)	0.19 (1.39)	0.2 (1.63)	0.19 (1.41)	0.2 (1.45)	0.19 (1.5)	0.2 (1.37)	0.2 (1.48)
Imageability^e^	4.34 (1.54)	4.33 (1.33)	4.33 (1.27)	4.34 (1.42)	4.33 (1.33)	4.32 (1.41)	4.32 (1.34)	4.32 (1.26)

a Frequency measures denote occurrences per million (CELEX; Baayen et al., 1993).

b Number of words in the CELEX database with the same bigram.

c Summed frequency of the words with the same bigram.

d Number of words of the same length which differ only by one letter.

e Võ et al. (2006); Zero denotes emotional neutrality—positive values denote positive emotional valence (max: +3); imageability ranges from 1 (low imaginability) to 7.

**Table A2:** **Means (standard deviations) of the characteristics of the pretarget words of the four experimental setups**.

	**Fixed-paced ERPs**		**Self-paced ERPs**		**Valid-preview FRPs**		**Masked FRPs**	
	**Old**	**New**	**Old**	**New**	**Old**	**New**	**Old**	**New**
	***M* (*SD*)**	***M* (*SD*)**	***M* (*SD*)**	***M* (*SD*)**	***M* (*SD*)**	***M* (*SD*)**	***M* (*SD*)**	***M* (*SD*)**
Frequency	13 (29)	12 (28)	12 (26)	12 (27)	12 (29)	12 (40)	12 (32)	13 (35)
Bigram count [N]	275 (398)	274 (303)	275 (300)	276 (393)	275 (300)	275 (302)	275 (261)	276 (322)
Bigram freq.	5572 (6537)	5569 (6970)	5582 (7032)	5567 (9274)	5581 (6652)	5578 (7585)	5564 (8540)	5577 (8053)
Syllables [N]	1.84 (0.37)	1.84 (0.37)	1.84 (0.37)	1.84 (0.37)	1.84 (0.37)	1.84 (0.37)	1.84 (0.37)	1.84 (0.37)
Letters [N]	6.04 (1.41)	6.04 (1.31)	6.04 (1.4)	6.04 (1.54)	6.04 (1.32)	6.04 (1.51)	6.04 (1.28)	6.04 (1.4)
Neighbors [N]	1.46 (2)	1.46 (2.09)	1.46 (1.56)	1.46 (2.18)	1.46 (1.73)	1.46 (2.07)	1.46 (2.57)	1.46 (2.21)

For a description of the measures see Table A1.
